# A Retrospective Audit of Demography and Different Surgical Modalities Adopted for Giant Cell Tumor of Bone in Eastern India

**DOI:** 10.7759/cureus.29520

**Published:** 2022-09-23

**Authors:** Indrajeet Kumar, Wasim Ahmed, Nishant Kashyap, Manish Kumar, Manish K Saw, Ravi Shekhar

**Affiliations:** 1 Orthopaedics, Indira Gandhi Institute of Medical Sciences, Patna, IND; 2 Surgical Oncology, Indira Gandhi Institute of Medical Sciences, Patna, IND; 3 Biochemistry, Indira Gandhi Institute of Medical Sciences, Patna, IND

**Keywords:** eastern india, surgical modalities, demography, giant cell tumor of bone, retrospective audit

## Abstract

Background and objective

There is scarce data on demography and different surgical treatment modalities for giant cell tumor of bone (GCTB) from eastern India. In light of this, the present study aimed to examine the demographic characteristics, different surgical treatment modalities, and recurrence rate of GCTB at a tertiary care institute in Bihar.

Materials and methods

A retrospective audit of 52 GCTB patients who were treated at the center from January 2016 to December 2020 was conducted. The minimum follow-up period was one year. GCTB patients underwent surgical procedures ranging from extended intralesional curettage with bone graft or bone cement with or without fixation to wide local excision to resection with or without reconstruction or amputation depending on the stage and site of the tumors.

Results

The mean age of patients was 31.86 years (range: 13-67 years). The distal femur (20 patients, 38.46%) and proximal tibia (11 patients, 21.15%) were the most common sites of the tumor. Sixty-eight confirmed cases (male: 32, female: 36) of GCTB were operated on, with a male-to-female ratio of 1:1.125. Sixteen patients (four males and 12 females) were lost to follow-up. So, the final study consisted of 52 patients with a median age of 28 years (first quartile: 24 years, third quartile: 38 years). The majority of patients (32 patients, 61.53%) were in the third and fourth decades of life.

Conclusion

Based on this retrospective audit, it is concluded that the knee region is the most common site of GCTB. Surgery is the mainstay of management. Most of the patients came under Campanacci Grade 3 with low compliance with follow-up and adherence to the treatment. Hence, educational programs, the establishment of early detection centers, and timely referral to expert treatment are necessary.

## Introduction

A giant cell tumor of bone (GCTB) is a benign, locally aggressive tumor with unpredictable biological behavior [1]. Most of the cases (80%) occur after the epiphyseal fusion between the third and fifth decades of life. Females are slightly more affected with a male-to-female ratio of 1:1.5 [2,3]. The tumor rarely undergoes malignant transformation and it occurs only in approximately 1% of cases [4-7]. It can occur before the age of 14 years in about 3% of cases, and only about 13% of cases occur beyond the age of 50 years [8]. These tumors are mostly located around the knee (distal femur, proximal tibia) [9]. The distal radius is the third most common site, but the condition has been reported in almost all other anatomical sites [1,9,10,11]. Swelling and activity-related pain are the most common presentations, which can progress to pain at rest. In rare cases, patients may remain relatively asymptomatic and may present with a pathological fracture [1].

The diagnosis of GCTB is based on radiological imaging in conjunction with confirmatory histology. Lesions are lytic and usually expansile, eccentrically located in the epiphysis extending into the metaphysis of long bones. The lesions sometimes have a soap-bubble appearance due to the ridging of the surrounding bone. Typically, there is no periosteal reaction, no clear zone of transition, and no marginal sclerosis [1,8].

Surgery is the mainstay of treatment. It depends on the Campanacci stage and the location of the tumor. The standard treatment has ranged from extended curettage with/without polymethylmethacrylate (PMMA) bone cement or bone graft to wide resection, and a wide range of results have been reported for all modalities [1,12]. This usually involves extended curettage with chemical adjuvants in the form of either liquid nitrogen or phenol or a high-speed burr and followed by filling of the tumor cavity with PMMA bone cement or bone graft or bone graft with biocomposite [8,12-17]. Wide local excision is recommended for cases where excision results in no significant morbidity, such as those involving proximal fibula and flat bone [12,17].

The local recurrence rate is very common among patients treated with curettage and the majority of recurrent cases are detected within three years of therapy [13,17,18]. Filling the cavity after the removal of the tumor mass reduces the risk of recurrence [13]. Although GCTB is categorized as a benign neoplasm, it may metastasize to the lungs in about 1-2% of cases eventually [19].

As most of the available literature on bone tumors is from western India [2] and southern India [12,20] and no literature is available on GCTB from the state of Bihar, the purpose of this retrospective audit was to report the demographic data, treatment, outcomes, and recurrence rates related to GCTB after various types of primary treatment and pulmonary metastasis. This retrospective review is the first attempt to provide demographic data on GCTB from the poor state of Bihar in eastern India.

## Materials and methods

**Figure 1:**
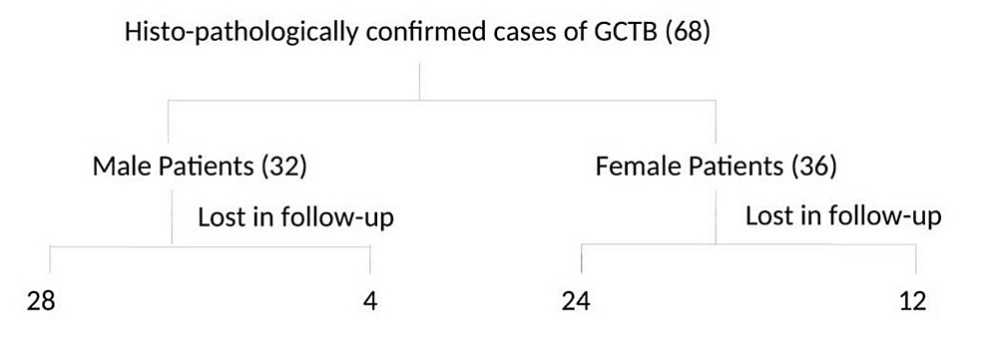
Flow diagram of inclusion/exclusion of patients GCTB: giant cell tumor of bone

We retrospectively analyzed inpatient cases of biopsy-confirmed GCTB treated at a tertiary center in eastern India from January 2016 to December 2020. Patient demographics, tumor sites and grade, treatment, and outcomes were evaluated. Approval from the Institutional Ethical Committee was obtained before the commencement of the study. The inclusion criteria were as follows: patients with histopathologically confirmed GCTB and a minimum follow-up of 12 months. The cases that did not undergo any kind of surgical intervention after histological confirmation were excluded from the study. Local X-rays and chest X-rays were done at every follow-up at six weeks, three months, six months, and 12 months, and thereafter at every six months. Figure 1 presents a flow diagram of the patient recruitment process.

X-ray of the involved part along with CT or MRI of the lesion, and chest X-ray or CT scan of the chest for evaluation of pulmonary metastasis were done. A bone scan was done in all cases to evaluate for multicentric sites of involvement. Treatments included extended intralesional curettage with phenol, extended curettage with bone graft or bone cement with or without fixation, wide local excision with or without reconstruction, and amputation depending on the site and extent of the disease.

## Results

**Figure 2:**
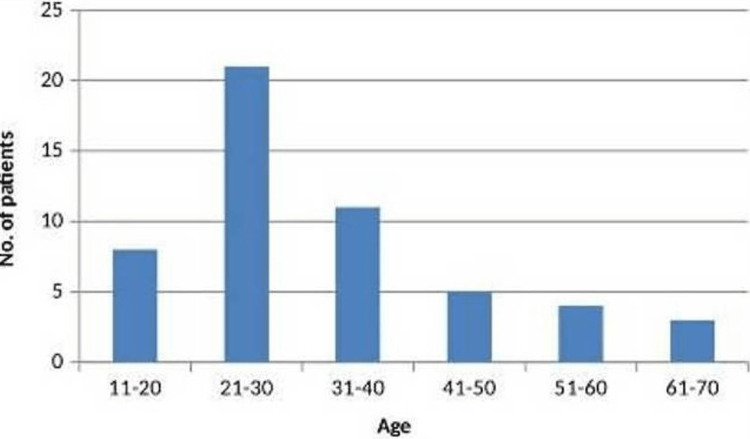
Age distribution in years

Sixty-eight confirmed cases (male: 32, female: 36) of GCTB were operated on, with a male-to-female ratio of 1:1.125. Sixteen patients (four males and 12 females) were lost to follow-up. So, the final study consisted of 52 patients (Figure 1) with a median age of 28 years (first quartile: 24 years, third quartile: 38 years). The majority of patients (32 patients, 61.53%) were in the third and fourth decades of life (Figure 2).

**Figure 3:**
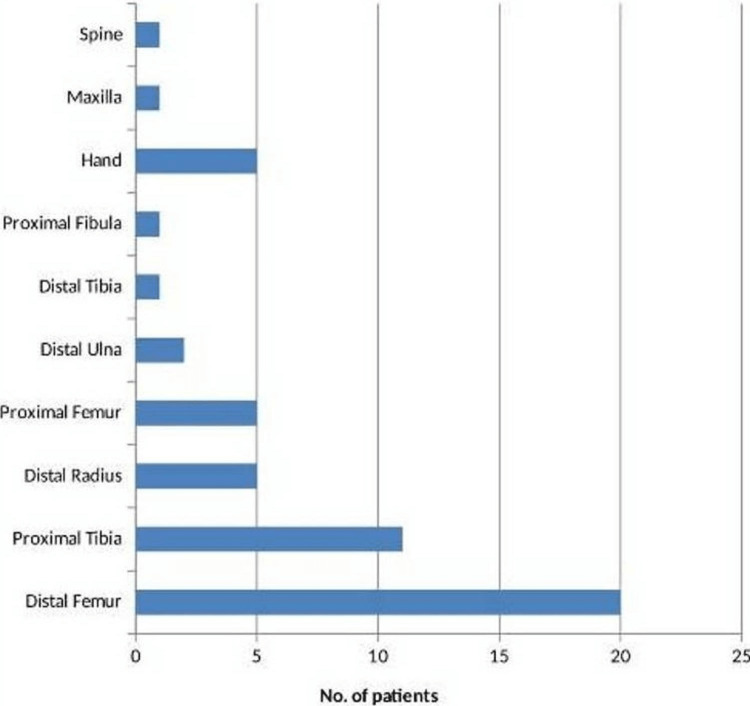
Location of tumor

The left side-to-right side ratio of tumor involvement was 1.04 (right side: 25, left side: 26). Distal femur (20 patients, 38.46%) and proximal tibia (11 patients, 21.15%) were the most common sites. Other sites were the distal radius, proximal femur, distal tibia, proximal fibula, distal ulna, metacarpal, phalanx, spine, and maxilla (Figure 3).

Five patients presented with pathological fractures. Radiologically, Campanacci grade 3 was found in 31 patients (59.61%) and grade 2 in 21 patients (40.38%). Surgical management was done in all patients (Table 1).

**Table 1:** Types of surgery

Types of surgery	Number of patients
Curettage + bone grafting	7
Curettage + bone cement	7
Curettage + bone graft + bio-composite	6
Curettage + bone graft + implant	4
Curettage + bone graft + bio-composite + implant	5
Curettage + bone cement + implant	4
Wide excision	3
Wide excision + reconstruction	5
Wide excision + mega prosthesis	6
Amputation	5

**Figure 4:**
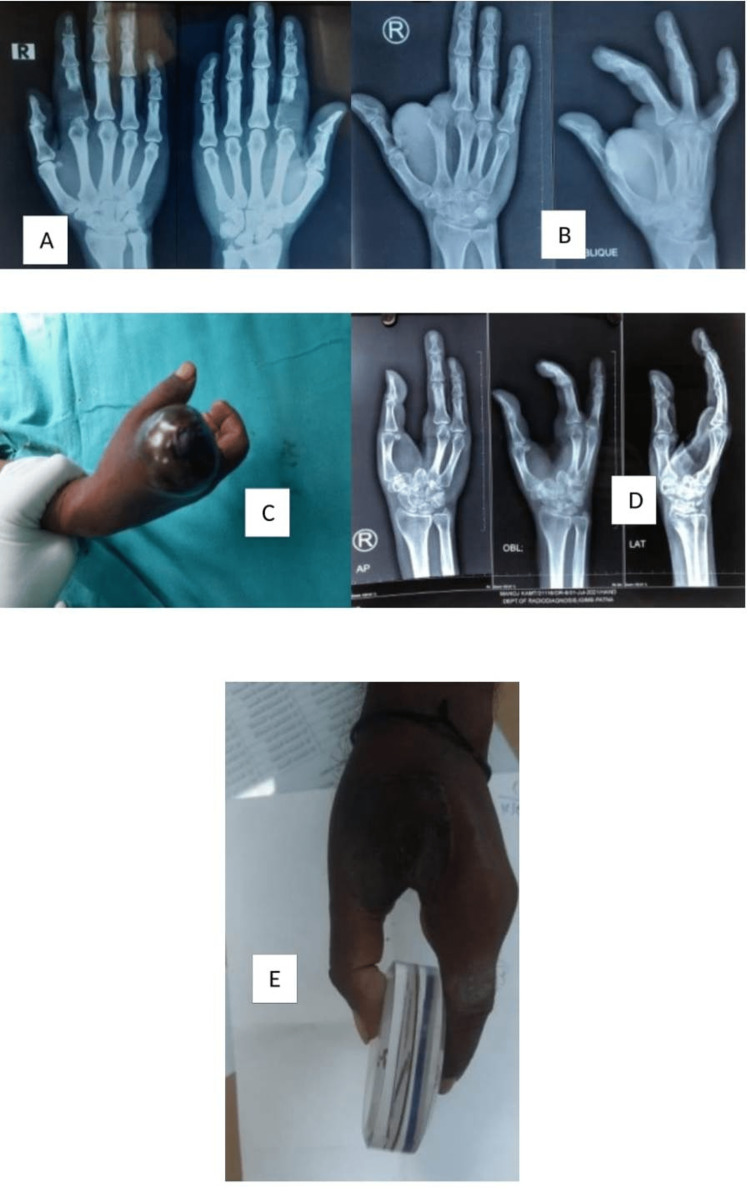
Ray amputation due to recurrence of GCTB in the proximal phalanx on the right side GCTB: giant cell tumor of bone

**Figure 5:**
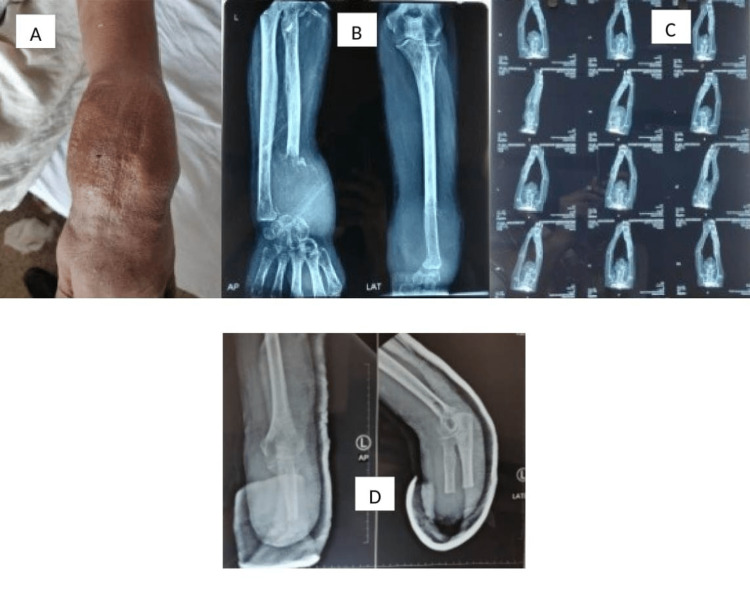
Below-elbow amputation for GCTB, distal end radius invading wrist joint with the erosion of carpal bone with carpal bone involvement and partially encasing radial artery at the wrist GCTB: giant cell tumor of bone

**Figure 6:**
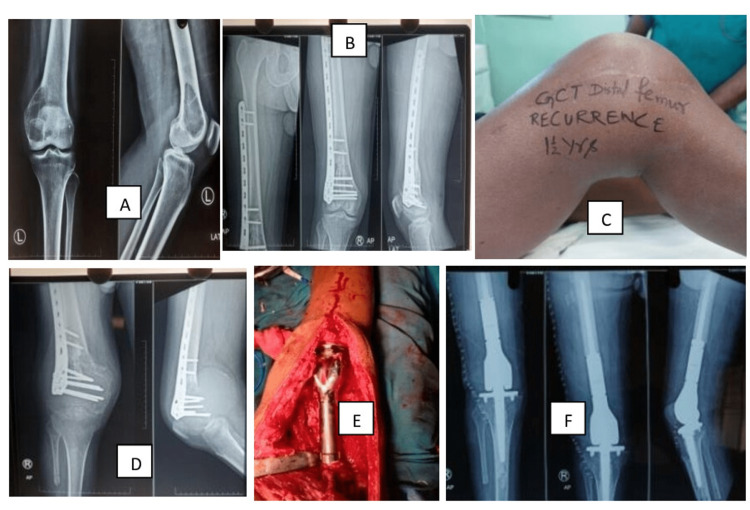
Reconstruction with megaprosthesis of GCTB distal femur, recurrence after 1.5 years GCTB: giant cell tumor of bone

Thirty-three patients were surgically managed with curettage with bone cement or bone graft with/without biocomposite, with or without implant fixation. Wide excision of the tumor was done in three cases of tumors of the distal end ulna and the proximal end of the fibula. Wide excision and limb reconstruction with ipsilateral fibula were done in five cases of GCT distal end of the radius. Excision of tumor and limb reconstruction with megaprosthesis were done in six cases for GCT of the proximal end femur and distal end femur. Amputation was done in a total of five cases. In one case, ray amputation was done for recurrence in the proximal phalanx; in one case, above-knee amputation was done for recurrence in the proximal tibia; in two cases, above-knee amputation was done for aggressive tumors presenting late. Infective ulcerative lesion of overlying skin was also seen in one case; in one case, below-elbow amputation was done for an aggressive tumor involving the radius, ulna, and carpal bone partially encasing the radial artery (Figures 4-6).

Phenol as local adjuvant therapy was used in 31 cases. We used C-arm in most of the cases to guide the extension of the curettage and avoid joint penetration. The minimum follow-up was 12 months (range: 12-48 months). In five of 52 patients, tumor recurrence was observed during the follow-up period. Lung metastasis was not found in any of the patients.

## Discussion

This audit documents the demographic information, tumor location, treatment, and outcomes of GCTB patients in Bihar state in eastern India. Most of the available literature showed a slight female predominance in GCTB [2,3,20]. A study by Jain et al. reported a male predominance at a tertiary care hospital in south India [21]. In our study, a male predominance was observed with respect to the number of patients included in the final analysis, with a female-to-male ratio of 1:1.16. Most of the patients in our study were between 20-40 years of age, which reflects the results of previous studies by Gupta et al. and Karpik et al [2,3].

In our study, 59.61% of tumors were found around the knee, 9.61% in the distal radius, and 9.61% in the proximal femur, which reflects the results of other studies in the literature [2,3]. GCTBs are mostly located at the ends of long bones and in about 50-65% of cases, the distal femur and proximal tibia are involved, with the distal radius being involved in about 10% of cases [9,10]. GCTB involving the hand, pelvis, ulna, and proximal fibula is rare [22,23]. Our results mostly matched with these reports, with 59.61% of tumors occurring around the knee, 9.61% in the distal radius, and 9.61% in the proximal femur. Bone resection is usually not advised because of morbidity that occurs after resection. It is indicated in the proximal radius, fibula, distal ulna, small bones of the hand and foot, coccyx, sacrum, and pelvic bones, where reconstruction is not possible.

Amputation was reserved for massive recurrence, or in malignant transformation where limb salvage surgery was not possible [12]. There was no case of malignant transformation in our study, but we performed amputation as the primary modality of treatment due to the delayed presentation of disease with skin ulceration and massive bony destruction in three cases. We did amputation in two cases of recurrence. Adjuvant radiation therapy was not used, based on studies regarding the efficacy of therapy and the risk of sarcomatous change after radiotherapy [24]. It can be used as an alternative to surgical options in cases that cannot be treated surgically or cases with severe disfigurement after surgery [25]. Radiotherapy was not used in any of the patients in our study. The rate of lung metastases in our study was 0%, which aligns with other studies in the literature, where lung metastases range from 0 to 4% [19,22]. In 2013, denosumab [humanized monoclonal antibody against the receptor activator of nuclear factor-κB-ligand (RANKL)] was approved for the management of advanced GCTB. However, we did not use denosumab or zoledronic acid in combination with residual cavity management strategies. Errani et al. found that the recurrence rate was significantly higher in patients treated with both curettage and denosumab compared to curettage alone [26]. Also, Tsukamoto et al. and Zhao et al. found that perioperative use of denosumab increases the risk of local tumor recurrence and it should be avoided [27,28]. Recent studies suggest that intravenous zoledronic acid as an adjuvant reduces tumor recurrence rates in surgically treated GCTB [29,30].

Our study has a few limitations. Our sample size was small, and we recommend studies with a greater number of cases (multicentric studies) and longer follow-ups to draw definitive conclusions. In our study, we found that in this part of India, there was a high rate of amputation: 3/52 as the primary modality for giant cell tumors, which is quite unique. We have not found any studies in the literature supporting such a high rate of amputation as the primary modality. This may be due to a lack of awareness, literacy, and poor socioeconomic conditions. Despite its limitations, this audit does, however, provide novel information about the demographics and different surgical modalities adopted for GCTB.

## Conclusions

Based on our findings, the knee region is the most common site of GCTB. Most of the patients belonged to Campanacci grade 3 with low compliance with follow-up and adherence to the treatment. Due to the late presentation of the disease, breach of skin and massive tumor size lead to a situation where limp salvation surgery is impossible even with a prosthesis, thereby resulting in mutilating surgery. Hence, there is a need to raise awareness through educational programs in rural areas and the establishment of centers with facilities for early detection, as well as referrals for expert treatment for avoiding mutilating surgeries.
